# Functionalized Nano-adsorbent for Affinity Separation of Proteins

**DOI:** 10.1186/s11671-018-2531-4

**Published:** 2018-05-30

**Authors:** Xueyan Zou, Fengbo Yang, Xin Sun, Mingming Qin, Yanbao Zhao, Zhijun Zhang

**Affiliations:** 1 Engineering Research Center for Nanomaterials, Henan University, Kaifeng, 475004 China; 2 National & Local Joint Engineering Research Center for Applied Technology of Hybrid Nanomaterials, Henan University, Kaifeng, 475004 China; 3 Collaborative Innovation Center of Nano Functional Materials and Applications of Henan Province, Henan University, Kaifeng, 475004 China; 4 Institute of Plant Stress Biology-State Key Laboratory of Cotton Biology, Henan University, Kaifeng, 475004 China

**Keywords:** Silica nanospheres, SnO_2_, Affinity separation, Peroxidase activity, Redox state

## Abstract

Thiol-functionalized silica nanospheres (SiO_2_-SH NSs) with an average diameter of 460 nm were synthesized through a hydrothermal route. Subsequently, the prepared SiO_2_-SH NSs were modified by SnO_2_ quantum dots to afford SnO_2_/SiO_2_ composite NSs possessing obvious fluorescence, which could be used to trace the target protein. The SnO_2_/SiO_2_ NSs were further modified by reduced glutathione (GSH) to obtain SnO_2_/SiO_2_-GSH NSs, which can specifically separate glutathione *S*-transferase-tagged (GST-tagged) protein. Moreover, the peroxidase activity of glutathione peroxidase 3 (GPX3) separated from SnO_2_/SiO_2_-GSH NSs in vitro was evaluated. Results show that the prepared SnO_2_/SiO_2_-GSH NSs exhibit negligible nonspecific adsorption, high concentration of protein binding (7.4 mg/g), and good reused properties. In the meantime, the GST-tagged GPX3 separated by these NSs can retain its redox state and peroxidase activity. Therefore, the prepared SnO_2_/SiO_2_-GSH NSs might find promising application in the rapid separation and purification of GST-tagged proteins.

## Background

The easy separation and purification of proteins are very important in xenobiotic biotransformation, drug metabolism, biosynthesis of prostaglandins and steroid hormones, and degradation of aromatic amino acids [1–6]. The separated proteins can be used for antigen and vaccine production, molecular immunology and structural, and biochemical and cell biological studies. Glutathione *S*-transferase (GST) represents a major group of detoxification isoenzymes which can be used in GST gene fusion system and medicine effect targeting field or as tumor markers [7, 8]. Various methods such as precipitation, chromatography, ultrafiltration, and dialysis are currently available for purifying various proteins, and in particular, affinity separation based on the natural biological affinity between biological macromolecules and complementary ligands is of extraordinary significance [9–14]. The successful production and purification of full-length, soluble, and natural fusion proteins, however, are still retarded by various obstacles such as the need for pretreatment to remove the cell debris and colloid contaminants, a relatively long operation time, and protein solubility. These drawbacks, fortunately, could be overcame by applying nanomaterials to assist the separation and purification of the target proteins [15–19]. For example, magnetic SiO_2_-NiO nanocomposite is capable of separating His-tagged proteins [20]. Nanomaterials, nevertheless, still have short legs in the separation of various proteins because they are often inactive to visualization and fluorescence techniques which can be used as sensitive biomolecular and medical diagnostic tools to combat biological warfare [21–23]. In this sense, it is imperative to find nanomaterials with fluorescent responses so as to promote their application in the separation and purification of recombinant protein like glutathione peroxidase 3 (GPX3).

We pay particular attention to nanoscale SnO_2_ quantum dots (QDs), because, as an n-type wide-bandgap (3.6 eV) semiconductor with good chemical stability and biocompatibility, SnO_2_ exhibits optical absorbance in visible spectral region. Herein, we establish a smart pathway to introduce fluorescent SnO_2_ QDs onto the surface of silica nanospheres (NSs), hoping to develop a desired SnO_2_/SiO_2_ nanostructure with potential application in the separation and purification of GST-tagged proteins. Firstly, thiol-functionalized silica nanospheres (SiO_2_-SH NSs) were prepared through a hydrothermal route. Resultant SiO_2_-SH NSs were compounded with SnO_2_ quantum dots to afford SiO_2_/SnO_2_ composite NSs possessing obvious fluorescence absorption. The SiO_2_/SnO_2_ NSs were further modified by reduced glutathione (GSH) to obtain SiO_2_/SnO_2_-GSH NSs with potential for the affinity separation of GST-tagged protein. The ability and the peroxidase activity of the prepared SnO_2_/SiO_2_-GSH NSs in separating GST-tagged proteins were evaluated by SDS-PAGE analysis.

## Experimental

### Material and Methods

Hexadecyltrimethyl ammonium bromide (CTAB), tin (IV) chloride (SnCl_4_), triethylamine (TEA), and isopropanol were provided by Tianjin Kermel Chemicals Reagent Company (Tianjin, China). AgNO_3_ was purchased from Tianjin Fuchen Technology Development Co., Ltd. (Tianjin, China). 3-Mercaptopropyl-trimethoxysilane (MPS) was offered by Alfa-Aesar (Shanghai, China). Tetraethyl orthosilicate (TEOS) was supplied by Tianjin Fuchen Chemicals (Tianjin, China). Dihydronicotinamide adenine dinuclectide phosphate (NADPH), thioredoxin, and thioredoxin reductase were obtained from Sigma (Beijing, China). Glutathione Sepharose 4B (Stockholm, USA) was from GE Healthcare. Dithiothreitol (DTT) was available from Aladdin Industrial Corporation (Inalco SPA, Italy). All chemical reagents were of analytical reagent and used without any further purification.

### Preparation of SnO_2_ Quantum Dots

In a typical synthesis [24], 3.5 g SnCl_4_·5H_2_O was added into 50 mL H_2_O, then 5 mL ammonia was added into the solution under stirring. Subsequently, the precipitation obtained by centrifugation was washed with deionized water for several times to remove the excessive Cl^−^ ions. Thirty milliliters of deionized water was added into the obtained precipitate, and then, the pH of the solution was adjusted to be 12 by 2 mol/L ammonia. The mixed solution was transferred into a Teflon-lined stainless steel autoclave, sealed and heated at 150 °C for 24 h. Upon completion of heating, the mixed solution was cooled, centrifuged, and fully washed with ethanol-isopropanol (volume ratio 1:1) to obtain SnO_2_ QDs.

### Preparation of SnO_2_/SiO_2_-SH NSs

In a typical synthesis, 0.2 g SnO_2_ QDs and 0.09 g CTAB were dissolved in the mixed solvent of H_2_O (42.5 mL) and absolute alcohol (7 mL) under magnetic stirring (200 G, *r* = 180 mm). Into resultant solution was added 2.7 mL TEA under additional 20 min of stirring. The mixed solution was heated at 60 °C for 5 h while 3.5 mL TEOS and 0.35 mL of MPS were slowly dropped, followed by centrifuging (12,800 G, *r* = 180 mm) and fully washing with HCl-ethanol (30 mL) and water (30 mL) to obtain SnO_2_/SiO_2_-SH NSs for three times, which was dispersed in water (0.12 g/mL).

### Surface Modification of SnO_2_/SiO_2_-SH NSs

Four milliliters of 0.12 g/mL SnO_2_/SiO_2_-SH NSs was washed with PBS (0.01 mol/L, pH = 7.4) for three times. These SnO_2_/SiO_2_-SH NSs were added into 30 mL 16.7 mg/mL GSH solution and oscillated at 37 °C for 24 h (120 rev/min) with a constant temperature oscillator. At the end of oscillation, the mixed solution was centrifuged to provide SnO_2_/SiO_2_-GSH NSs; then, the precipitate was fully washed with 30 mL PBS (0.01 mol/L, pH = 7.4) for three times to remove excessive GSH via physical adsorption, thereby affording desired SnO_2_/SiO_2_-GSH NSs. The resultant SnO_2_/SiO_2_-GSH NSs were added into alcohol (25%, *v*/*v*) and stored at 4 °C.

### Separation of GST-Tagged Proteins

The mixed proteins were collected from the cell lysate of *Escherichia coli*, which is by water lysis (concentration 0.01 mol/L, pH 7.4). For in vitro protein expression, the protein region containing the coding sequence of glutathione peroxidase 3 (GPX3, amino acids 37–206), Open stomata 1 (OST1), and ABA insensitive 2 (ABI2, amino acids 100–423) in *Arabidopsis thaliana* was cloned and inserted in frame into the plasmid pGEX-6p1 (GPX3 was used as control). pGEX-GPX3, pGEX-OST1, and pGEX-ABI2 constructs were introduced into *E. coli* BL21 (DE3) cells. The recombinant GST-tagged proteins were purified using Glutathione Sepharose 4B and SnO_2_/SiO_2_-GSH NSs. The primers used for cloning the genes were as follows: for GPX3, forward primer, 5′- GATGGATCCTCGCCATCGACGGTGGAACAA-3′; reverse primer, 5′- CACCTCGAGTCAAGCAGATGCCAATAGCTT-3′; for OST1, forward primer, 5′- GCCGAATTCATGGATCGACCAGCAGTGA-3′; reverse primer, 5′- CCCGTCGACTCACATTGCGTACACAATC-3′; for ABI2, forward primer, 5′- GCGGAATTCGAGAGTAGAAGTCTGTTTG-3′; reverse primer, 5′- GCGCTCGAGTCAATTCAAGGATTTGCTC-3′.

After being washed with PBS solution (0.01 mol/L, pH = 7.4), the prepared SnO_2_/SiO_2_-GSH NSs were directly introduced into 1000 μL *E. coli* lysate and shaken at 4 °C for 2 h (rotation speed: 90 rev/min) to allow the SnO_2_/SiO_2_-GSH NSs to capture GST-tagged proteins. Upon completion of shaking, these NSs were isolated from the solution by centrifugation and fully washed with PBS solution to remove any residual uncaptured proteins. The GST-tagged protein-bound SnO_2_/SiO_2_-GSH NSs were washed with 300 μL and 0.5 mol/L GSH solution for three times to disassociate GST-tagged proteins from their surface. Separately collected protein solutions were detected by sodium dodecylsulfate polyacrylamide gel electrophoresis (SDS-PAGE). The concentration of the separated proteins was determined by BCA protein Assay Kit. The SnO_2_/SiO_2_-GSH NSs can be reused to separate the target proteins for several times by the same method.

### Measurement of Glutathione Peroxidase Activity

The separated GPX3 activity was measured by the spectrometric determination of NADPH consumption at 340 nm as described by Delaunay et al. [25]. The GST tag was cut off by PreScission protease from GST-tagged GPX3, and then, the GPX3 was used for activity analysis. Firstly, 98 μL reaction buffer solution (including 100 mmol/L Tris-Cl, 0.3 mmol/L NADPH, 1.34 μmol/L thioredoxin, and 0.18 μmol/L thioredoxin reductase from *E. coli* lysate) was added into a tube; after mixing completely, 1.35 μmol purified GPX3 was added into the resultant reaction buffer solution. Then, the mixed solution was added into 2 μL H_2_O_2_ (5 mmol/L) to initiate the reaction and NADPH consumption at 340 nm was collected by the spectrometric determination.

### Analysis of Redox States of Purified GPX3

The GST tag was cut off from GST-tagged GPX3 by PreScission protease. The separated GPX3 was treated with 5 mmol/L H_2_O_2_ and 1 mmol/L DTT for 10 min to change the redox states of the purified GPX3. The resultant GPX3 was used for in vitro analysis of redox states. Extracts were evaluated by nonreducing 15% SDS-PAGE gel.

### Characterization

The morphology and composition of the prepared SnO_2_/SiO_2_-GSH NSs were analyzed by transmission electron microscopy (TEM, JEM-2010, Japan), scanning electron microscopy (SEM, JSM 5600LV, Japan), X-ray diffraction (XRD, X’ Pert Philips, Holland), and fluorescence spectrometer (FL, FluoroSENS, Britain, at the excitation wavelength of 260 nm). The separated GST-tagged proteins were detected with sodium dodecylsulfate polyacrylamide gel electrophoresis (SDS-PAGE, Power PAC 300, China), with the preconcentration voltage of 70 V and the separation voltage of 120 V. The constant temperature oscillator was from Shanghai ChemStar Instruments, Co., Ltd. (ATS-03M2R, China). The concentration of the separated proteins was determined by BCA protein Assay Kit (Beijing CoWin Biotech, China).

## Results and Discussion

### TEM, SEM, XRD, and Fluorescent Analyses of SnO_2_ QDs and SnO_2_/SiO_2_-GSH NSs

**Fig. 1:**
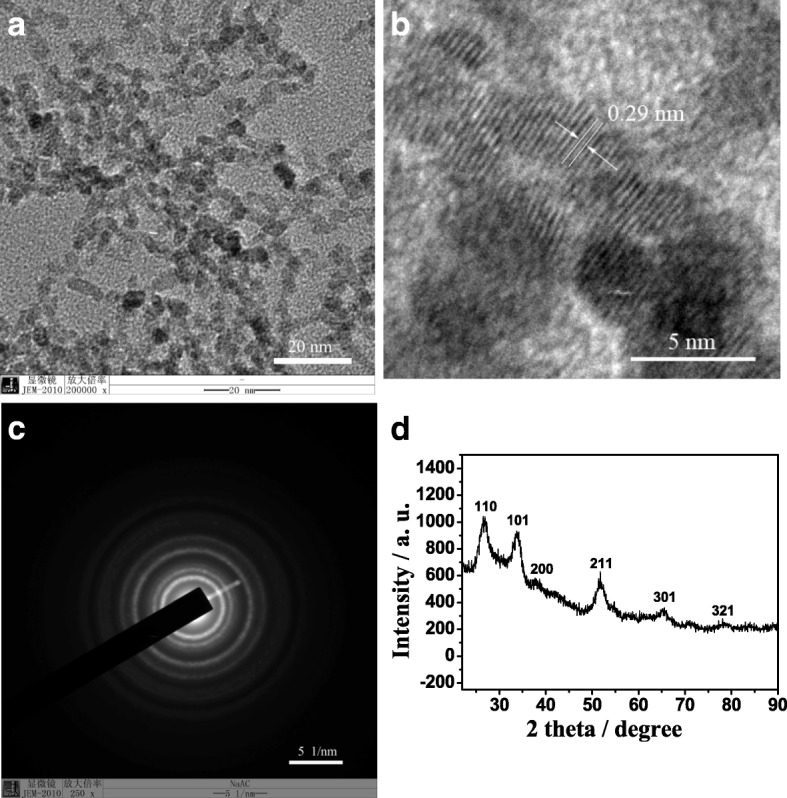
TEM (**a**), HRTEM (**b**) images, selected area electron diffraction pattern (**c**) and XRD pattern (**d**) of prepared SnO_2_ QDs

 Figure 1 gives the high-resolution TEM (HRTEM) images and XRD pattern of the synthesized SnO_2_ QDs. It can be seen that the synthesized SnO_2_ QDs are of spherical shape and have an average diameter of 5 nm, which exhibits a narrow particle size distribution (Fig. 1a), and their lattice spacing of (110) plane is 0.29 nm (Fig. 1b). The well-resolved lattice image demonstrates that the prepared SnO_2_ QDs have a highly ordered crystalline structure. Corresponding selected area electron diffraction pattern of SnO_2_ QDs (Fig. 1c) can be indexed to a single Cassiterite phase, which is consistent with the relevant XRD pattern (Fig. 1d). Namely, the characteristic peaks at 2 theta = 26.6° (110), 33.9° (101), 38.0° (200), 51.8° (211), 65.9° (301), and 78.7° (321) are consistent with the standard XRD data of Cassiterite SnO_2_ (JCPDS card no. 41-1445). Besides, the intense XRD peaks indicate that the prepared SnO_2_ QDs are well crystallized, and the absence of other characteristic peaks suggests that they do not contain hematite or hydroxide impurities.

**Fig. 2:**
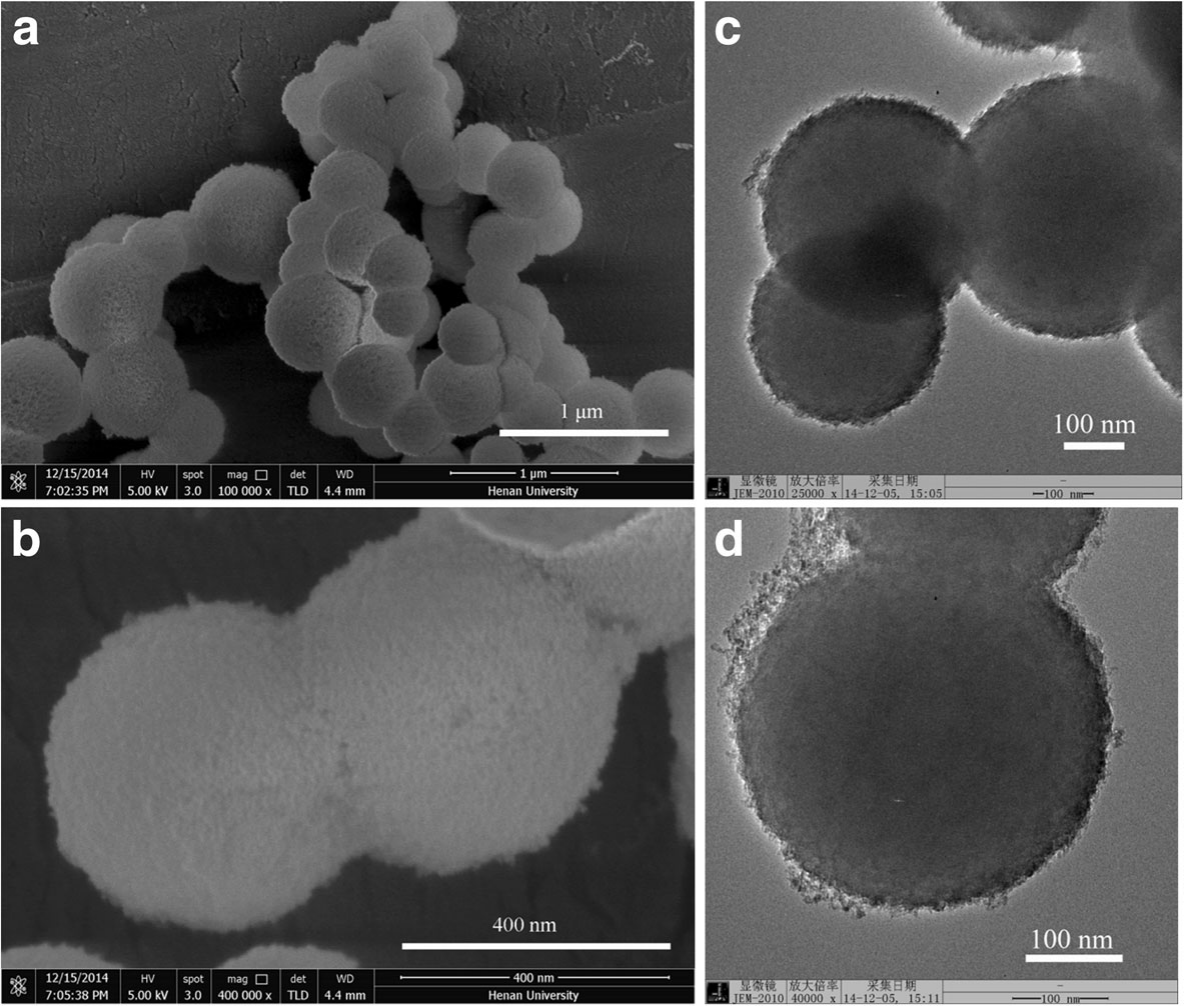
SEM (**a**, **b**) and TEM (**c**, **d**) images of the prepared SnO_2_/SiO_2_-GSH NSs

 Figure 2 gives the SEM and TEM images of SnO_2_/SiO_2_-GSH NSs. It can be seen that the prepared SnO_2_/SiO_2_-GSH NSs are of a spherical shape and have an average diameter of about 430 nm, and their surface seems to be somewhat rough (Fig. 2a, b). In the meantime, it can be seen that the SnO_2_ QDs (about 5–15 nm) are modified on the surface of SiO_2_ microspheres (Fig. 2c, d), which is consistent with the corresponding SEM images. It is indicated that the SnO_2_ and silica NSs have been aggregated.

**Fig. 3:**
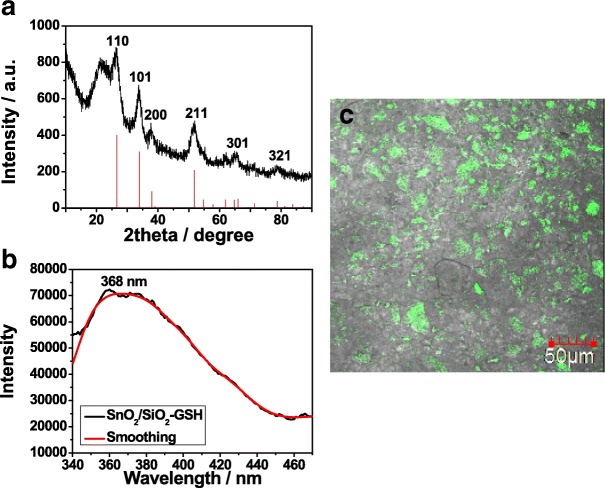
XRD pattern (**a**), fluorescence spectrum **(b**), and fluorescence imaging (**c**) of prepared SnO_2_/SiO_2_-GSH NSs

Figure 3a gives the XRD pattern of the synthesized SnO_2_/SiO_2_-GSH NSs. The major peaks at 2 theta = 110°, 101°, 200°, 211°, 301°, and 321° are consistent with those of SnO_2_ (Fig. 1d). Besides, SnO_2_/SiO_2_-GSH NSs show an intense characteristic peak of amorphous silica around 23° (JCPDS card no. 76-0933), which indicates that SnO_2_ possessing visible light response has been successfully introduced onto the surface of SiO_2_ NSs. Figure 3b shows the fluorescent spectrum of SnO_2_/SiO_2_-GSH NSs at 368 nm. It can be seen that SnO_2_/SiO_2_-GSH displays intense fluorescent emission, which is attributed to oxygen vacancies of SnO_2_. Figure 3c gives the fluorescence imaging of SnO_2_/SiO_2_-GSH NSs when these NSs are used to separate GST-tagged GPX3 in *E. coil* lysate. It can be seen that there are obvious green fluorescence where the prepared SnO_2_/SiO_2_-GSH NSs are used. It indicates that SnO_2_ was modified on the surface of SiO_2_ and the SnO_2_/SiO_2_-GSH NSs have good fluorescence properties.

### SDS-PAGE Analysis

**Fig. 4:**
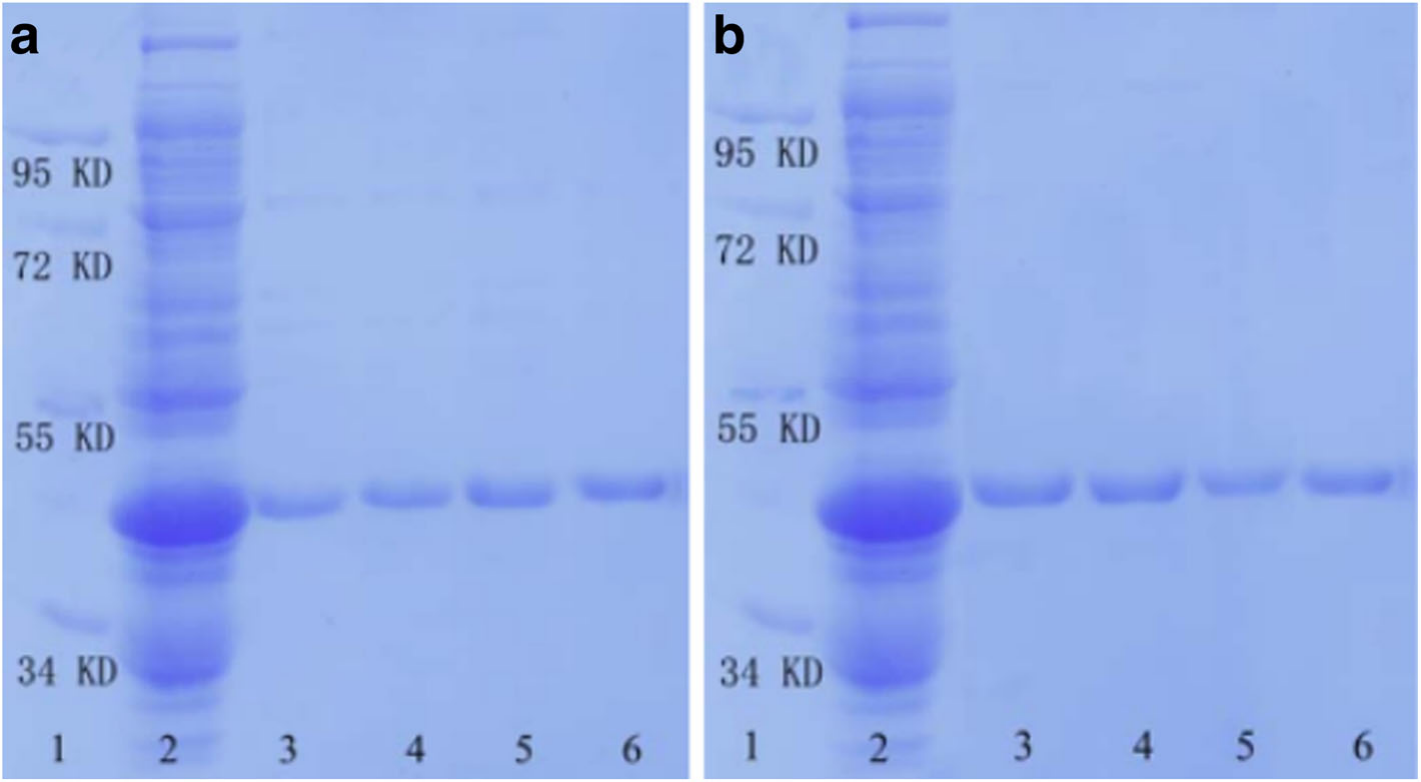
SDS-PAGE analysis of purified GST-tagged proteins separated by SnO_2_/SiO_2_-GSH NSs. **a** Lane 1, marker; lane 2, *E. coli* lysate; lanes 3–6 refer to the fractions washed off from the SnO_2_/SiO_2_-GSH NSs with different concentrations of GSH solution (lane 1, 10 mmol/L; lane 2, 20 mmol/L; lane 3, 50 mmol/L; lane 4, 100 mmol/L). **b** Lane 1, marker; lane 2, *E. coli* lysate; lane 3, 1st separation; lane 4, 2nd separation; lane 5, 3rd separation; and lane 6, the fractions washed off from the Glutathione Sepharose 4B

To estimate the ability of the prepared SnO_2_/SiO_2_-GSH NSs in separating GST-tagged proteins, we conducted SDS-PAGE analysis. Figure 4 shows the SDS-PAGE analysis result of the GST-tagged GPX3 separated by SnO_2_/SiO_2_-GSH NSs. It can be seen that the SnO_2_/SiO_2_-GSH NSs can efficiently enrich target proteins from *E. coli* lysate, and in particular, the quantity of the disassociated proteins tends to increase with incremental concentration of GSH in the range of 10–100 mmol/L (lanes 3–6 in Fig. 4a). It is clear that the target proteins can be separated specifically by the prepared SnO_2_/SiO_2_-GSH NSs from the *E. coli* lysate and there was hardly any nonspecific.

In order to investigate the reused properties of the prepared SnO_2_/SiO_2_-GSH NSs, we repeatedly used them to separate GST-tagged GPX3. As shown in Fig. 4b (lane 1 refers to the marker, lane 2 refers to GST-GPX3-contained *E. coli* lysate, lane 3 refers to 1st separation, lane 4 refers to 2nd separation, lane 5 refers to 3rd separation, and lane 6 refers to the fractions washed off from Glutathione Sepharose 4B), the synthesized SnO_2_/SiO_2_-GSH NSs exhibit special selectivity towards GST-tagged GPX3 extracted from *E. coli* lysate, and their specificity and affinity remain unaffected after three cycles of repeat separation.

**Fig. 5:**
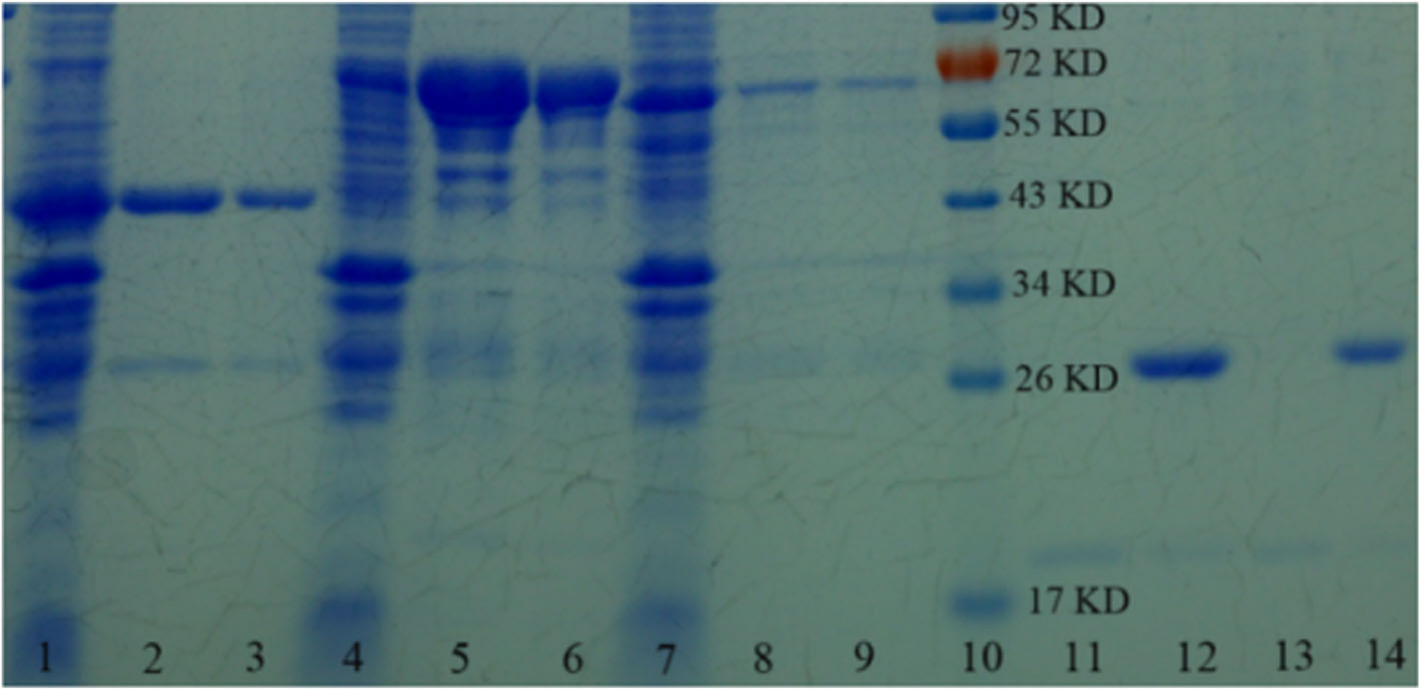
SDS-PAGE analysis of the purified recombinant GPX3, OST1, and ABI2 proteins. Lanes 1, 4, and 7, *E. coli* lysate; lanes 2, 5, and 8, the proteins eluted from commercial Glutathione Sepharose 4B (GE Healthcare, USA); lanes 3, 6, and 9, the proteins eluted from SnO_2_/SiO_2_-GSH NSs; lane 10, the marker; lanes 11 and 13, GPX3 obtained after the GST tag is cut off from Glutathione Sepharose 4B bound GST-GPX3 and SnO_2_/SiO_2_-GSH NSs bound GST-tagged GPX3; lanes 12 and 14, GST tag eluted from Glutathione Sepharose 4B and SnO_2_/SiO_2_-GSH NSs

To test the universality of the synthesized SnO_2_/SiO_2_-GSH NSs for purifying GST-tagged proteins, we selected three kinds of GST-tagged proteins (GST-tagged GPX3, GST-tagged OST1, and GST-tagged ABI2) to conduct experiments. As shown in Fig. 5, GST-tagged GPX3, OST1, and ABI2 proteins can be separated specifically by SnO_2_/SiO_2_-GSH NSs from the *E. coli* lysate (lanes 3, 6, 9); then, we can get both GPX3 (cut off the GST tag from SnO_2_/SiO_2_-GSH NSs binding GST-tagged GPX3) and GST tag eluted from SnO_2_/SiO_2_-GSH NSs (lane 13, the GPX3; lane 14, the GST tag), which has a similar effect with Glutathione Sepharose 4B (lanes 2, 5, 8, 11, 12). The concentrations of purified proteins by SnO_2_/SiO_2_-GSH NSs were 7.4 mg/g (GST-tagged GPX3), 7.1 mg/g (GST-tagged OST1), and 6.8 mg/g (GST-tagged ABI2), which indicate that SnO_2_/SiO_2_-GSH NSs are good to purify GST-tagged proteins from the *E. coli* lysate. In order to compare the binding capacity between the prepared SnO_2_/SiO_2_-GSH NSs and the other material, Glutathione Sepharose 4B (purchased in Stockholm, USA) was used as comparison experiment material. The total proteins purified by Glutathione Sepharose 4B were 7.1 mg/mL (GST-tagged GPX3), 6.9 mg/mL (GST-tagged OST1), and 5.6 mg/mL (GST-tagged ABI2), respectively. It can be seen that the binding capacity of the prepared SnO_2_/SiO_2_-GSH NSs is higher than that of commodity 4B.

### Analysis of Redox State and Peroxidase Activity of GST-Tagged GPX3

**Fig. 6:**
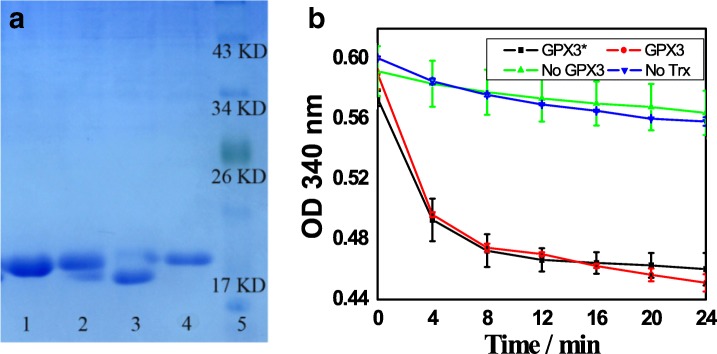
**a** In vitro assays of GPX3 redox state: lanes 1 and 3 (the oxidized GPX3) and 2 and 4 (the reduced GPX3) refer to GPX3 obtained after the GST tag is cut off from Glutathione Sepharose 4B and SnO_2_/SiO_2_-GSH bound GST-tagged GPX3, respectively; lane 5, marker. **b** Assays of peroxidase activity of GPX3

In order to analyze the redox state and activity of GST-tagged GPX3 separated by the prepared SnO_2_/SiO_2_-GSH NSs, we cut off the GST tag to obtain the separated GPX3. Figure 6a shows given in vitro assays of GPX3 redox state (corresponding to a representative gel from three independent experiments). Lanes 1 and 2 refer to GPX3 obtained after the GST tag is cut off from Glutathione Sepharose 4B bound GST-tagged GPX3 (lane 1 is the oxidized GPX3 and lane 2 is the reduced GPX3); lanes 3 and 4 refer to GPX3 obtained after the GST tag is cut off from SnO_2_/SiO_2_-GSH bound GST-tagged GPX3 (lane 3 is the oxidized GPX3 and lane 4 is the reduced GPX3); lane 5 refers to the marker. As shown in Fig. 6a, the purified GPX3 separated from Glutathione Sepharose 4B (lanes 1 and 2) and SnO_2_/SiO_2_-GSH NSs (lanes 3 and 4) have the oxidized and reduced states, and the reduced GPX3 migrates more slowly than the oxidized counterpart. This is well consistent with our previous findings that GPX3 is present in oxidized and reduced states in vitro, and its reduced and oxidized forms can be separated as a result of modification of the reduced Cys residues [26–28].

Figure 6b shows the assays of peroxidase activity of GPX3: line GPX3*, complete assay of the purified GPX3 in the presence of Glutathione Sepharose 4B, thioredoxin, thioredoxin reductase, NADPH, and H_2_O_2_; line GPX3, complete reaction among SnO_2_/SiO_2_-GSH NSs separated GPX3, thioredoxin, thioredoxin reductase, NADPH, and H_2_O_2_; line No GPX3, complete reaction in the absence of GPX3; and line No Trx, complete reaction in the absence of thioredoxin. Figure 6b shows the glutathione peroxidase activity of the purified GPX3 separated from Glutathione Sepharose 4B and SnO_2_/SiO_2_-GSH NSs in vitro. It can be seen that, with thioredoxin as the substrate, the purified GPX3 exhibits significant peroxidase activity, which indicates that the GPX3 separated from SnO_2_/SiO_2_-GSH NSs is existent in the natural state.

## Conclusions

A facile method is established to fabricate silica-protected SnO_2_ QD nanospheres (SnO_2_/SiO_2_ NSs). The SnO_2_/SiO_2_ NSs are further modified by glutathione to afford SnO_2_/SiO_2_-GSH NSs for the affinity separation of glutathione *S*-transferase-tagged (GST-tagged) recombinant protein. Findings indicate that, in terms of the ability to separate GST-tagged GPX3, GST-tagged LOV, and GST-tagged ABI2, the prepared SiO_2_/SiO_2_-GSH NSs exhibit specific separation, high concentration of protein binding, and good reused properties. Besides, the GPX3 separated from the GST-tagged GPX3 retains its redox states in vitro and GPX activity as well, which means that the prepared SnO_2_/SiO_2_-GSH NSs might have a promising potential for the rapid separation and purification of GST-tagged proteins.

## Abbreviations

ABI2: ABA insensitive 2
CTAB: Hexadecyltrimethyl ammonium bromide
DTT: Dithiothreitol
GPX3: Peroxidase activity of glutathione peroxidase 3
GSH: Reduced glutathione
GST-tagged: Glutathione *S*-transferase-tagged
MPS: 3-Mercaptopropyl-trimethoxysilane
NADPH: Dihydronicotinamide adenine dinuclectide phosphate
OST1: Open stomata 1
QDs: Quantum dots
SDS-PAGE: Sodium dodecylsulfate polyacrylamide gel electrophoresis
SEM: Scanning electron microscopy
SiO_2_-SH NSs: Thiol-functionalized silica nanospheres
SnCl_4_: Tin (IV) chloride
TEA: Triethylamine
TEM: Transmission electron microscopy
TEOS: Tetraethyl orthosilicate
XRD: X-ray diffraction
